# Tuberculous Orbital Abscess Associated with Thyroid Tuberculosis

**Published:** 2011-07

**Authors:** Kumudini Sharma, Vikas Kanaujia, Anu Jain, Sukhdeep Bains, Suvarna Suman

**Affiliations:** Department of Neuro-Ophthalmology, Sanjay Gandhi Post Graduate Institute of Medical Sciences, Lucknow, India

**Keywords:** Orbital Tuberculosis, Cold Abscess, Thyroid Tuberculosis

## Abstract

**Purpose:**

To report an unusual presentation of tuberculosis.

**Case Report:**

A six-year old boy presented with left upper lid swelling of 15 days’ duration and an asymptomatic midline neck mass from 2 months ago. Imaging studies, and microbiologic tests which demonstrated acid-fast bacilli in the fine needle aspirate of the thyroid mass, both confirmed a diagnosis of cold tuberculous thyroid abscess with presumed hematogenous spread to the orbit. The patient demonstrated marked improvement of both lesions with antitubercular drugs.

**Conclusion:**

This case illustrates a very rare association of orbital and thyroid tuberculosis.

## INTRODUCTION

Involvement of orbital tissues by tuberculosis (TB) is a rare entity which may lead to formation of a cold abscess. TB remains the leading cause of death due to infectious disease worldwide.^1^ In western Europe and the United States, there has been a resurgence of the disease since 1985. This increase is mostly due to TB in immigrants who account for 40 to 50% of all cases in the western world.^2^ In developing countries such as India, despite familiarity with bizarre manifestations of TB, cases such as the patient presented herein pose a diagnostic challenge. Diverse presentations of orbital tuberculosis have been reported in the literature including abscess formation, scrofuloderma, and periostitis with bone erosion. However, orbital involvement due to hematogenous spread from a primary thyroid lesion, which is a rarity in itself, is very unusual.

## CASE REPORT

A 6-year-old boy presented to the neuro-ophthalmic clinic with swelling over the lateral aspect of his left eye for 15 days. The swelling was not painful, but had been progressive and associated with lacrimation and drooping of the eyelid. Concurrently, an asymptomatic midline swelling was evident over the patient’s neck which had been present for 2 months. A history of occasional low-grade fever and a decrease in appetite were also reported. There was no history of bleeding tendency or similar swellings elsewhere, neither of cough or hemoptysis.

On examination, visual acuity was 6/6 in the right eye and 6/24 (6/6 with pinhole) in the left. The left upper eyelid demonstrated fullness over its lateral aspect with erythema of the overlying skin. A minimally mobile, non-pulsatile, non-compressible, diffuse and firm mass with mild tenderness was palpable in the lateral upper lid. There was mild ptosis and the eyeball was displaced inferomedially. Both the palpebral and bulbar conjunctivae were congested. Anterior and posterior segments in both eyes were normal. The pupils were 4 mm in diameter, with brisk direct and consensual light reactions and no relative afferent defect. The neck swelling was soft, fluctuant, non-tender and moved on deglutition.

**Figure 1:**
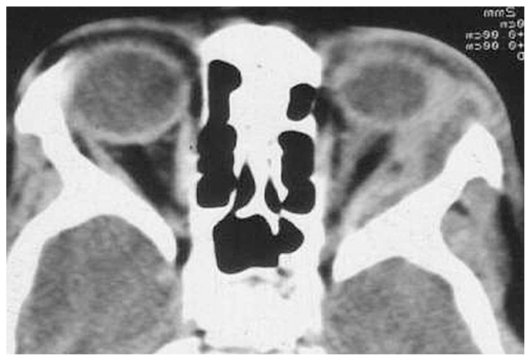
Contrast-enhanced computed tomography depicting a ring-enhancing, low density lesion in the lateral extraconal space of the left orbit. Note medial displacement of the globe and lateral rectus.

Ultrasonography (US) of the orbital lesion showed low reflectivity, while contrast-enhanced computed tomography demonstrated a ring-enhancing, low density lesion in the lateral extraconal space with medial displacement of the globe and lateral rectus (Fig. 1). The lacrimal gland could not be separately visualized. Sections obtained from the brain were normal.

Laboratory investigations revealed hemoglobin level of 9.3 g/dL, leukocyte count of 15,000 cells/mm^3^, and erythrocyte sedimentation rate of 48 mm in the first hour. Thyroid function tests demonstrated high levels of thyroid stimulating hormone (TSH), 25.75 milliunits per liter (normal, 0.3 to 5.0 mU/L), with T_3_ and T_4_ within standard limits. US of the neck revealed a cystic swelling measuring 3 × 2 cm in the left lobe of the thyroid gland. A technetium-99 scan was performed which revealed a hypofunctioning nodule involving the lower third of the left lobe, extending to the isthmus.

**Figure 2:**
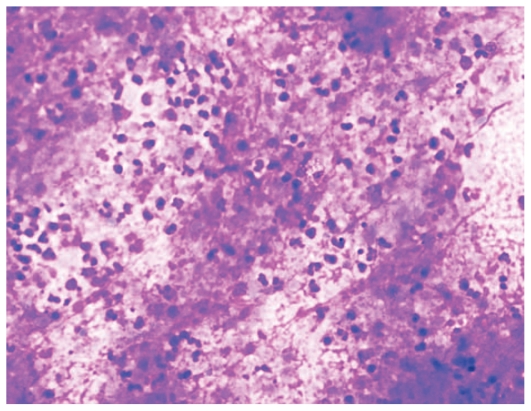
Photomicrograph of fine needle aspirate obtained from the thyroid mass demonstrates degenerated polymorphonuclear cells, lymphocytes, and epitheloid cells in a necrotic background (May-Grünwald Giemsa staining, magnification ×100)

US-guided fine needle aspiration of the orbital lesion was performed and the specimen underwent microscopic examination, and culture for bacteria, mycobacteria and fungi. Cytology demonstrated lymphocytes and a few giant cells, but no micro-organisms. Cultures remained sterile after 2 weeks. Fine needle aspiration from the neck mass revealed acid-fast bacilli, together with lymphocytes, and epitheloid and giant cells (Fig. 2). Mycobacteria grew on the culture media incubated with the thyroid specimen. Chest X-ray and abdominal US were normal.

**Figure 3:**
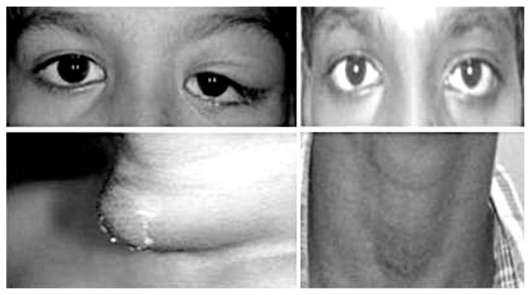
Orbital and neck swelling (left images) resolved completely with antitubercular treatment. Pictures on the right were taken 4 years after complete resolution of the condition.

Eventually, a diagnosis of thyroid gland TB with coexisting tuberculous orbital abscess was made and the patient received a 3-drug antitubercular regimen. The orbital and neck swelling resolved after 6 months of therapy and the child remained asymptomatic up to one year (Fig. 3).

## DISCUSSION

Although several reports of orbital TB have been published since it was first described by Abadie in 1881^3^,^4^, the condition is still rare, even in endemic regions.^5^ Orbital TB generally occurs due to hematogenous dissemination or contiguous spread, and is often unilateral.^4^ Patients of any age may become affected, but the condition typically occurs in the first two decades of life.^6^ Orbital TB has five presentations: classical periostitis, orbital soft tissue tuberculoma or cold abscess without bony involvement, orbital TB with bony involvement, spread from paranasal sinuses, and tuberculous dacryoadenitis.^7^ The ocular adnexa, including the nasolacrimal system and overlying skin, may also become involved.

The primary tuberculous focus is most frequently pulmonary. However extra-pulmonary involvement, such as lymphadenopathy or abdominal disease, may be the main presentation in some instances. To establish a diagnosis of orbital TB, evidence of active or inactive systemic TB should be sought. Orbital TB has been reported in individuals with no pulmonary TB but with manifestations of the disease in other areas, such as tuberculous sinusitis and constrictive pericarditis.^8^

Primary or secondary TB of the thyroid gland is an extremely rare condition even in countries with endemic TB; only isolated reports and a small number of case series have been described in the literature.^9^ Customary teaching states that the thyroid gland, like the pancreas and striated muscles, is resistant to TB. This has been attributed to high vascularity, rich lymphatics, strong capsules, cellular paucity, and enhanced phagocytosis, as well as the bactericidal effect of colloid and iodine.^10^ When thyroid TB does occur, it can present as multiple thyroid lesions associated with miliary TB, solitary caseating thyroid nodules, cold abscesses, chronic fibrosing inflammation, and acute abscesses.^10^ In most cases, thyroid TB is secondary to disease occurring in other primary locations.^9^ In such cases, the thyroid is affected by spread of bacilli via hematogenous or lymphogenous routes, or directly from laryngeal or cervical lymphadenitis.^11^ Primary thyroid TB is difficult to diagnose.^12^ Diagnosis is based on a positive tuberculin test, caseating granulomatous inflammation on histopathology and positive culture of *Mycobacterium tuberculosis* if a specimen is obtained early in the course of the disease.^9^

Our patient who was unaware of the thyroid nodule, presented because of orbital swelling. The absence of signs of acute inflammation prompted us to consider TB as the cause of the condition. Fine needle aspiration cytology from the abscess showed chronic inflammatory cells, however acid-fast staining and culture were both negative. Even in the presence of histological features typical for TB, acid-fast bacilli are usually not found in orbital lesions^4^ which was also the case in our patient. Initially, the thyroid nodule was not considered as the primary tuberculous focus until fine needle aspiration cytology was positive for TB and the culture grew tuberculosis bacilli as well. Evidence of clinical or biochemical thyroid dysfunction has seldom been described with thyroid TB,^11^ but our patient had grossly elevated TSH levels.

In summary, the patient described herein was a peculiar case of primary tuberculous thyroid abscess with hematogenous spread to the orbit. We believe that the differential diagnosis of a peripherally enhancing lesion in the orbit should include tuberculous cold abscesses, especially in endemic areas. Awareness of rare manifestations of TB is of utmost importance because the incidence of TB is increasing worldwide due to HIV infection and immigration of people from endemic areas to the West.
